# “We try our best to offer them the little that we can” coping strategies of Ghanaian community psychiatric nurses: a qualitative descriptive study

**DOI:** 10.1186/s12912-020-00449-3

**Published:** 2020-06-23

**Authors:** Frederick Yaw Opare, Patience Aniteye, Agani Afaya, Nathaniel Glover-Meni

**Affiliations:** 1 Department of Nursing, School of Nursing and Midwifery, University of Health and Allied Sciences, Ho, Ghana; 2 Department of Community Health Nursing, School of Nursing and Midwifery, University of Ghana, Legon, Ghana; 3 Department of General and Liberal Studies, University of Health and Allied Sciences, Ho, Ghana

**Keywords:** Community health, Psychiatric, Nursing, Coping, Stress, Religion, Self care

## Abstract

**Introduction:**

Community psychiatric nurses work in extremely stressful environments with intense patient relationships as they try to prevent self-harm and manage aggressive behaviors. In order to improve their ability to manage the stressful work environments, community psychiatric nurses need to incorporate formal coping strategies into their daily work routines. With evidence-based coping strategies, community psychiatric nurses can effectively manage the stressful situations in their work environment to increase their work longevity. The purpose of this study was to explore the individual coping strategies currently used by community psychiatric nurses in practice in order to develop an intervention strategy for future implementation.

**Methods:**

This was an exploratory qualitative study using an interpretative approach. A purposive sampling method was used to identify participants from the community psychiatric nurses in a region of Ghana. Participants were recruited and interviewed, guided by semi-structured questions, until saturation was reached. The interviews were audio-taped, transcribed verbatim, and analyzed thematically.

**Results:**

A total of 13 participants, 10 women and 3 men ages 26 to 60 years, were interviewed for this study. From the inductive analysis, four coping themes emerged from the data including: 1) self-disguise, 2) reliance on religious faith, 3) self-motivation, and 4) reduction in the number of home visits. The participants described their work environment as stressful, almost to the point of overwhelming. In this regard, they identified the individual coping strategies as critical daily practices for self care to manage their high stress levels.

**Conclusion:**

Individual coping strategies are often used by community psychiatric nurses in daily practice. The participants identified personal coping strategies as critical interventions to manage stress and to decrease their risk for burnout. However, community psychiatric nurses must develop.

personal-mastery in various coping strategies to care for themselves, as well as motivate them despite the challenging working environment. The individual coping strategies adopted by community psychiatric nurses was not only helped them deliver care, but also protected their clients so people would not label them as ‘mental patients.’ Collectively, the four strategies reported in this study need to be developed into a cohesive and comprehensive intervention.

## Introduction

Globally, mental health services have focused on delivering holistic recovery orientated care within the community. The mainstay of professionals to promote and maintain these are the community psychiatric nurses (CPNs) [1]. Nursing is a stressful profession [1–10] and is associated with significant pressure, because of the demanding, challenging, and stressful nature of the professional responsibilities [4, 7, 11]. CPNs manage excessive stress resulting from workloads, hazardous situations, and role conflict; especially with inadequate social and professional support when delivering mental health care at the community level [1, 4, 12, 13]. Consequently, CPNs should adopt strategies to cope with work related stress in order to avoid feeling overwhelmed and experiencing burnout. These strategies, called “coping strategies,” are how psychiatric nurses overcome stressful and difficult circumstances. The “coping strategies” are defined as a set of cognitive and behavioral approaches used by individuals to handle specific situations (internal or external) [14].

Due to the strenuous work environment, psychiatric nurses need to maintain good physical and mental health and to initiate techniques of coping that will strengthen their ability to cope effectively with stress [4, 7]. This, in turn, will reduce the levels of stress and burnout they experience [10]. Therefore, understanding how CPNs cope with job-related stress is an important workplace strategy, not only for the nurses themselves but also for the organisations they work for and eventually the patients who are receivers of their care [4].

CPNs across the globe, including Ghana, are faced with numerous challenges/stressful situations which in-turn affects them mentally, psychologically, and physically. These challenges include; lack of transportation, assaults by patients, shortage of staff, the stigma associated with mental illness, low morale, lack of resources, lack of feedback, intense interpersonal involvement, difficulties in nurse relationships and the ability to work together, poor supervision and non-compliance to medications by the patients, leading to patient relapse and readmissions [3, 4, 9, 10, 12, 15]. There is very little evidence about the various coping strategies these professionals adapt to effectively manage stress in the face of providing mental health care to patients in the communities amid these numerous challenges they confront. The purpose of this study was to explore the individual coping strategies currently used by community psychiatric nurses in their daily practice to inform the development of an intervention strategy for future implementation.

## Methods

### Research design

This exploratory qualitative research design used an interpretative approach [16] to gain an in-depth understanding of the coping strategies employed by CPNs when delivering care in the communities within the Accra metropolis. This design was selected to understand the meaning of experiences related to nursing practice and research [17].

### Research team and reflexivity

All interviews were conducted by FYO, who is a psychiatric nurse and a researcher. PA and AA are both nurses and researchers with professional interest in community psychiatric nursing. NGM is a research fellow with interest in community psychiatric nursing. One of the authors (PA) had extensive previous experience with qualitative research at the masters and PhD studies and supervised the interviews.

### Study setting and population

The study was conducted in the Accra metropolis which is the political and administrative authority for the city of Accra. The data were collected within January 2013. The target population for the study was CPNs working in the community psychiatric units and polyclinics in the Accra metropolis. About 60% of CPNs in Ghana work in Accra and all the CPNs in Accra metropolis meet weekly for a case conference.

### Inclusion and exclusion criteria

The inclusion criteria for the study were all CPNs who had graduated from the Psychiatric Nurses Training College and had more than three years of working experience. Community psychiatric nurses who refused to consent to participate where exempted from the study. Also, CPNs who were on leave were excluded from the study.

### Selection of participants

A purposive sampling method [18] was employed to recruit 13 participants from 6 sub-metro districts (Ayawaso, Ashiedu Keteke, Okaikoi, Lekma, Ablekuma, and Osu-Klottey) in Accra. Participants were recruited through a regional in-service training department of the Accra Psychiatric hospital for all CPNs in the region. At least 2 participants were selected from each of the 6 sub-metro districts.

### Interview guide

A semi-structured interview guide was used for data collection. The purpose of using a semi-structured interview guide was to generate in-depth and sincere data that will lead to a deeper understanding of the issues [19]. The interview guide was developed by reviewing related literature on coping strategies by CPNs [9–12, 20, 21] to gain insight into the phenomenon under study before the interviews. The interview guide consisted of participants’ demographic characteristics and questions on coping strategies (Interview guide added as additional file). The principal questions asked were; *“What do you do to overcome the challenges that affect your work? How do you cope with the challenges that affect you?”* The interview guide was pre-tested with 5 participants outside the study area to ensure that it measured its intended purpose. Interviews were audiotaped with the participants’ consent. Field notes of emotional expressions were taken consisting of salient points and observations of non-verbal communications were noted during the interviews. Restructuring of the interview guide was made after the pre-test for clarity and simplicity. A co-researcher and expert in psychiatric nursing reviewed the interview guide to determine its relevance to achieve the aim of the study.

### Data collection

Individual interviews were conducted each day for 13 days at a side office of the Accra Psychiatric hospital to ensure privacy during interviews. Face-to-face in-depth interviews were conducted and also, probing questions were used when needed to elicit more information from the participants. The interviews lasted between 30 and 60 min and were tape-recorded. Each interview session had a moderator and an assistant moderator. The moderator led the interviews and kept the discussion flowing. The assistant moderator operated the audio tape recorder and took comprehensive notes, and also responded to unexpected interruptions. All tape-recorded files, transcripts, and informed consent forms were stored anonymously in a secured password-protected digital storage system by FYO. Data saturation [22] was reached at the 12th participants. There was no repeated interview in this study.

### Data analysis

Inductive thematic analysis [23] was used to analyse data collected based on the objectives of the study. The audio recordings were transcribed verbatim by FYO. To ensure the accuracy of transcription, the transcripts were returned to participants for feedback and possible corrections were made. The transcripts were read and reread by three (FYO, AA, PA) of the authors to ensure acquaintance with the data. The audio recordings were frequently referred to, to enable easy interpretation of the responses in their actual context. The transcripts were coded by FYO and supervised by PA and the differences in coding discussed to ensure intercoder reliability and to reach an agreement with coding. The coding was started bottom-up and expanded and built on during each additional interview. After seven interviews, interim thematic template analysis was done and interim themes were generated which guided the subsequent interviews. The final coding was agreed upon by FYO and PA after all codings had completed. The data were manually analysed by authors.

### Trustworthiness

Different approaches were adopted to ensure trustworthiness which included the use of the same interview guide throughout the entire study. An audit trail was kept for researchers to validate the methods/steps undertaken in the study. To ensure the transferability of the study findings to a similar context a detailed description of the study setting, methodology (COREQ criteria were used) [24], and background of the sample have been provided. Furthermore, in-depth interviews ensured a full exploration of the CPNs coping strategies during healthcare delivery in the communities. Concurrent data analysis ensured that the CPNs coping strategies were further explored in subsequent interviews. To ensure validity, data were returned to participants (8) to crosscheck and verify their responses. Finally, the reporting of this study adhered to the 32 criteria recommended by the consolidated criteria for reporting qualitative research (COREQ) [24].

### Ethical considerations

The Institutional Review Board of Noguchi Memorial Institute for Medical Research of the University of Ghana, Legon approved the study (NMIMR-IRB CPN 022/12–13). Approval letters were submitted to the administrators of all of the community psychiatric units of the various health facilities. Verbal and written consent was obtained from all participants before individual interviews. Interviews were audio-recorded, with the approval of each participant. Participants were assured of confidentiality.

## Results

### Characteristics of study participants

Three (3) males and ten (10) females participated in the study, with their ages ranging from 26 to 60 years. All of them were CPNs with various academic and professional qualifications. Six (6) of the participants were registered mental health nurses, with bachelors’ degrees from the university. Three (3) were registered mental health nurses and the remaining four (4) were enrolled, mental health nurses.

Four main themes were derived from the data collected which included; reducing stigma by CPNs; reliance on religious faith, self-motivation by CPNs, and reduction in-home visits.

### Self-disguise (wearing mufti)

Most of the participants (10 out of 13) were stigmatized because they cared for patients who were mentally ill. The poor perceptions and attitudes that society holds about mental illness caused the CPNs a great deal of stress. Yet, the CPNs were committed to their work and employed several strategies to withstand the negative attitudes of community members.

In the interest of the patients and the CPNs themselves, they hid their identity from the public during home visiting. This was also to ensure privacy and confidentiality, as well as to ward off gossip about their clients. The disclosure was based on the discretion of the CPNs and was only done when it was necessary. Most of the participants said that they disguised themselves during home visits as a strategy to mitigate the effects of the stigma associated with the care of mentally ill patients. A participant narrated:

“*… society brands them as mad people; which our clients detest this attribution so much. Not wearing uniforms when visiting clients is a way of providing privacy. So when I go in mufti, no one recognizes me as a nurse …*” (Participant 2).

A 60-year-old CPN shared her reasons for not wearing a uniform:

*“Our reason for wearing mufti is because of the negative thought about mental illness. The clients or relatives feel ashamed that nurses in uniform do visit them because of their condition. For us CPNs, we protect our clients such that people might not say that there is a ‘mad person’ in this house and psychiatric nurses are following him or her …*” (Participant 3).

All participants (13) agreed that hiding their identity during home visiting of mentally ill patients was a way of protecting the privacy of their patients and making their clients comfortable. Mentally ill or so-called “mad people” are not respected in the Ghanaian society and therefore CPNs reportedly wore mufti rather than uniforms so that community members do not necessarily know why the person was being visited.

A CPN shared her rationale for hiding her identity:

“*… We do not wear uniforms because we don’t want our identity to be disclosed to the community members … we think it is the stigma attached to mental illness and that is why some of us do that”.* (Participant 6).

Some of the participants (9 out of 13) highlighted additional measures they took to further ensure that no one knew that their clients were patients who had mental health problems. This was done to avoid stigmatization. One of the measures was to make sure they knew exactly where their patients lived so that they avoided asking people. Not all streets in Ghana were labelled at the time of this study and it was a common practice to ask people for direction when one could not find his or her bearings. Participant 9 described her strategy to locate her patients:

*“We don’t wear uniforms when we are going on home visits because of the stigma. We wear only mufti. What we normally do, is that when we find our clients especially on Tuesdays at the Accra Psychiatric hospital, we try to find out if we have clients within our catchment area, then we take their landmarks for purposes of home tracing … we even take telephone numbers. This, in turn, helps us to get to the client’s house without asking for direction from strangers...”.*

Almost all the CPNs (12 out of 13) used landmarks to locate the houses of their clients. They also collected and kept telephone numbers of relatives so they could ask for directions from relatives, and not community members. Furthermore, they did not wear their name tags, but rather kept them concealed in their bags and only showed them to relatives of their clients.

### Reliance on religious faith

Some of the participants (7 out of 13) said that they used religious beliefs to console themselves concerning the challenges they encountered in their work. The CPNs trusted God as the source of grace, help, and abundance and suggested He worked as a mediator. The CPNs reported that they did all they could to help the patients and expected that God would reward them:

*“But the bible says give and it shall be given unto you. So, sometimes we take consolation that when we give … our Father also sees us through the challenges we grapple within our lives. So we sacrifice amid the challenges of delivering care to our patients knowing God will reward us …*” (Participant 1).

Another participant shared her belief in God:

*“Our work is demoralizing, the challenges are there and by the grace of God we are managing and we contain them”.* (Participants 10).

### Self-motivation

Despite the multitude of challenges faced by the CPNs, they continued to care for the mentally ill in their various communities. Participants (13 out of 13) were self-motivated to meet the demands of the work though they were faced with hazardous work environments such as slaps and attacks by patients. The CPNs reported that they were not recognized for the work they do, even though they thought their work comes with dangers and weariness. They were reportedly demoralized for the lack of recognition and job satisfaction. Even though some of them felt sad and hurt, they could not complain because in their view no one gave them a listening ear. They tried to ignore their inner sentiments of sadness and moved on with their work. Some were invigorated seeing their clients doing well. Others hoped that one day their work would be appreciated.

“*… if you have that kind of heart … if you are the tolerant type, you can continue to work amid attacks (slaps) by your patients. You can’t say because the client slapped me or the client poured urine on me, I won’t go and deliver care to him/her. At times you will have to motivate yourself, hoping that the care you render will improve the clients’ condition …*” (Participant 12***).***

Others expressed negative emotions regarding their job as CPNs but continued to render care by encouraging themselves:

*“It is painful but all the same we have taken it as our job so we try our best to do it.”* (Participant 6).

Another participant said:

*“Well, I have not regretted that I have done this work and have gotten into this condition. I’m happy that God has given me life to continue. I accept the situation as it is in the meantime and hope that something better will come someday … when I wake up every day I motivate myself”.* (Participant 9).

Participant 5 hoped to be noticed one day since she continued to do her best in terms of educating the public and conducting home visits to check on clients. She indicated, however, that she was happy that some of the clients were in good health:

*“We are still striving, still doing our part and hope to be noticed one day. I’m still doing my best through education, visiting my clients. So, I take it cool and I’m happy at least some of my clients are doing well”.* (Participant 5).

### Reduction in-home visits

Participants (8 out of 13) mentioned that sometimes they had to reduce the number of home visits as a means of dealing with the challenges they encountered in terms of financial and human resource constraints and lack of transport:

*“What we usually do is to restrict the number of visits that we embark on normal working days due to financial constraints. But we do increase the number of visits when we have student nurses around … because we have had a lot of students so we can do more visits within a short time frame …*” (Participant 12).

One participant noted:

*“Yes, yes if you are supposed to visit about 10 clients a day and there is no money, we can only do about 4 or 5 because that is how far the money can take you and it brings about reduction in-home visits and subsequently it is the client that suffers”.* (Participant 4).

A participant said:

*“At times, it’s like you draw your itinerary with regards to what you want to do with the clients but because there is no T & T, you have to leave this and cover a different thing because the means are not there for you to get to that place because, at the end of the day, you are supposed to work”.* (Participant 11).

## Discussion

The findings of this study highlight various forms of coping strategies adopted by Ghanaian CPNs working within the Accra metropolis. They adopted the following categories of coping strategies; Self disguise (wearing mufti), reliance on religious faith, self-motivation, and a reduction in the number of home visits for the client.

### Self-disguise (wearing mufti)

Wearing mufti was a coping strategy employed by CPNs in managing some difficulties encountered in their practice. Some of the CPNs mentioned that they wore mufti (casual wear) to visit clients as a disguise or a cover up of their profession. CPNs wore mufti due to the negative thoughts about mental illnesses in most Ghanaian settings and the stigmatization of the mental health persons and family caregivers [25]. Not wearing a uniform was also a way of protecting clients from stigmatization. A recent study in Ghana reported that clients and professionals were stigmatized because of the uniforms the professionals wore [25]. Not wearing a uniform in this study was a way of assuaging any bad feelings of the relatives who might misconstrue the nurses’ intentions for visiting them. This was also an attempt to reduce public suspicion and stigma towards the client and the family. In other words, this current finding is different from the study findings of Corbiere, Samson, Villotti, and Pelletier, which sought to provide a more complete and exhaustive perspective on the whole range of potential strategies to fight stigma in Canada by considering the perspectives of different stakeholders [26]. Corbiere, Samson, Villotti, and Pelletier, in their study, identified 15 categories of strategies to combat stigma and one was sharing/encouraging disclosure. The results from their study highlighted the need to pay more attention to the concept of disclosure of mental disorders in the process for de-stigmatization [26], but the current study’s findings sought not to disclose client identity to reduce stigma at the community level. The disparity in the study findings might be due to different study environments, cultural beliefs, and understanding of the mental illness. We, therefore, recommend public education on mental health and mental illness to be intensified. This is believed to provide rightful and adequate information on mental illness and health.

### Self-motivation

Participants also indicated that self-motivation was a coping strategy they used to deal with the demands of their work. Some participants stated that they were sometimes assaulted by patients but continued to do their work. Other participants mentioned that they did not have the desire of going for home visits these present times unlike when they started work as CPNs. Community psychiatric nurses continued to motivate themselves to deliver the best form of care they could even though they faced several challenges. Our study finding is similar to that of Wang, Kong, and Chair, who described three methods frequently used by nurses to cope with stress at their workplace; one of the methods used was optimistic (positive mindset, positive attitude, positive associations) [27]. The current study participants were truly optimistic to deliver care to their clients no matter the challenges. They had that positive inner drive known as self-motivation. The current study finding is also congruent with findings of Bonsu and Salifu-Yendork [25], where professionals (including CPNs) reported only being intrinsically motivated, considering the current state of mental health system in Ghana. Much of their inner drive was informed by their knowledge and achievements. Existing research by Roberts, Asare, Mogan, Adjase, and Osei, [28] also supports the current study finding, where they indicated that there were instances where professionals got motivated by the outcome of their patients’ recovery. It is realized that most of the CPNs in the current and previous studies engaged in positive problem-focused coping: where they develop the ability to strategize to address the reason for the stress. This coping strategy is considered to be the most effective way of dealing with workplace stress [10]. This implies that CPNs continue to offer health services despite the constraints of their work through intrinsic motivation.

### Reduction in-home visits

It was also found in this study that a reduction in the number of home visits was another measure CPNs adopted to deal with the challenges of their work. Some participants mentioned that because of financial constraints they sometimes regulated the number of home visits needed to be done. Some CPNs mentioned that they were always overwhelmed with the number of communities they were supposed to cover. They were overstressed due to the shortage of community psychiatric nurses in the region. Findings from Fagin et al., showed that psychiatric nurses used an inappropriate approach to managing stress by unconsciously or consciously distancing themselves from the cause of stress [21]. This finding by Fagin et al. [21] is consistent with the current study's finding where CPNs reduced the number of home visits due to stress. This coping strategy of reducing home visits or distancing themselves from sources of stress has negative repercussions on the therapeutic relationship between the psychiatric nurse and the clients [21]. The lack of CPNs will probably pose a major challenge to deliver adequate care in Ghanaian communities. Human resources are often cited as a major barrier to scaling up mental health services in low and middle-income countries [29]. The number of psychiatric nurses was estimated at 2.47 per 100,000 and probably the majority of them will be concentrated in the hospitals but not in the communities or rural areas [29]. We recommend that the ministry of health collaborates with the Nursing and Midwifery Council to setup more psychiatric training institutions. This will help scale up the number of psychiatric nurses in Ghana and further improve care delivery.

### Reliance on religious faith

The present study finding revealed participants used religious beliefs as a coping mechanism to console themselves concerning the challenges they encounter in their workplace. The CPNs trusted God as the source of grace, help, and abundance and suggested He worked as a mediator amid a challenging work environment. The current study disagrees with Bonsu, Salifu, and Yendork [25], where the mental health professionals (including nurses) never used religion as a coping mechanism amidst of the job stress. But the current study supports the findings of Salaree-Zareiyan, Ebadi, and Salaree [30], where nurses used religion as a coping strategy to mitigate a stressful work environment. The participants reported that nursing care was a religious duty, so they worked for spiritual reward than financial gains. Therefore, they do not feel tired because the objective is to satisfy God. Another study in South Africa reported that nurses relied on spiritual support to cope with their job stress while caring for mentally ill patients [7].

### Limitations of the study

The study engaged CPNs in only Accra metropolis within the Greater Accra Region of Ghana which constitutes a relatively small proportion in the whole region. Coping strategies adopted by CPNs in the Accra metropolis might differ among CPNs in other parts of the region and Ghana as a whole. Given this, future studies should consider using a larger sample that is representative of CPNs across the entire region, or nationwide. That notwithstanding, firstly, the study identified diverse coping strategies adopted by CPNs to avert stress and to improve mental healthcare in the Ghanaian communities. Secondly, the full iterative cycle for reporting qualitative studies was completed. After 7 interviews an initial data analysis was conducted, based on which the interview guide was improved for subsequent interviews, the researchers reverted to the field for new interviews to be conducted until data saturation was achieved. Finally, another strength of this study is its critical reflection on validity, where feedback from the study participants (8) is a representative sample of the study population.

## Conclusion

Individual coping strategies are often used by community psychiatric nurses in daily practice. The participants identified personal coping strategies as critical interventions to manage stress and to decrease their risk for burnout. However, community psychiatric nurses must develop personal-mastery in various coping strategies to care for themselves, as well as motivate them despite the challenging working environment. The individual coping strategies adopted by community psychiatric nurses not only helped them to deliver care, but also protected their clients so people would not label them as ‘mental patients.’ Collectively, the four strategies reported in this study need to be developed into a cohesive and comprehensive intervention.

## Supplementary information

**Additional file 1.** In-depth interview guide.

## Data Availability

The datasets used during this study are available from the corresponding author on a reasonable request.

## Abbreviations

CPNs: Community Psychiatric Nurses
