# Supplementary material for: “We try our best to offer them the little that we can” coping strategies of Ghanaian community psychiatric nurses: a qualitative descriptive study
Source: BMC Nurs. 2020 Jun 23;19:56. doi: 10.1186/s12912-020-00449-3 (PMC7310546; doi:10.1186/s12912-020-00449-3)
Supplement: Supplementary file 1 — Additional file 1. In-depth interview guide. [file 12912_2020_449_MOESM1_ESM.docx]

**In-depth interview guide**

**Coping strategies of Ghanaian Community Psychiatric Nurses working in the Accra Metropolis: A Qualitative descriptive study**

Start time: __ __ __ __ and End time __ __ __ __

Interviewer ID __ __ __ __

Interview date: __ __/__ __/____ DD / MM / YY

**Step 1:** Introduce yourself to the participant. Describe the purpose of the interview and how information will be used. Obtain oral/written consent.

**Step 2:** Ask the participant to identify herself and answer socio-demographic information prior to beginning the interview.

**Step 3:** Conduct the interview. Please remember to audio record the interview

**Section A**

**Demographics Characteristics**

Age

Sex

Marital Status

Religion

Level of Education

Number of children

Residence

**Section B**

**Focal Question**

*Please can you tell me about your experiences as a community psychiatric nurse in the discharge of your duty?*

1. How would you describe your work as a community psychiatric nurse?
2. How is it like caring for the mentally ill in the community as a nurse?
3. What perceptions does the society have about you as a community psychiatric nurse?
4. What are some of the support you need to render effective services to your clients/ family?
5. Please can you share with me the challenges you face as a community psychiatric nurse in the discharge of your duty?
6. *How do the challenges affect you as an individual? How do the challenges affect your work as a community psychiatric nurse?*
7. *What do you do to overcome the challenges that affect your work?*
8. *How do you cope with the challenges that affect you?*

Do you have anything else to share with me?
